# Stem cells isolated from human dental pulp and amniotic fluid improve skeletal muscle histopathology in *mdx*/SCID mice

**DOI:** 10.1186/s13287-015-0141-y

**Published:** 2015-08-28

**Authors:** Alessandra Pisciotta, Massimo Riccio, Gianluca Carnevale, Aiping Lu, Sara De Biasi, Lara Gibellini, Giovanni B. La Sala, Giacomo Bruzzesi, Adriano Ferrari, Johnny Huard, Anto De Pol

**Affiliations:** Department of Surgical, Medical, Dental and Morphological Sciences with interest in Transplant, Oncology and Regenerative Medicine, University of Modena and Reggio Emilia, via del Pozzo 71, 41124 Modena, Italy; Stem Cell Research Center, Department of Orthopaedic Surgery, University of Pittsburgh, 450 Technology Drive, Bridgeside Point II, Suite 206, 15219 Pittsburgh, PA USA; Department of Obstetrics and Gynecology, Arcispedale Santa Maria Nuova, viale Risorgimento 80, 42123 Reggio Emilia, Italy; Oro-Maxillo-Facial Department, AUSL Baggiovara, via Giardini 1355, 41126 Modena, Baggiovara Italy; Department of Biomedical, Metabolic and Neuroscience, University of Modena and Reggio Emilia, Children Rehabilitation Special Unit, IRCCS Arcispedale Santa Maria Nuova, viale Risorgimento 80, 42123 Reggio Emilia, Italy

## Abstract

**Introduction:**

Duchenne muscular dystrophy (DMD), caused by a lack of the functional structural protein dystrophin, leads to severe muscle degeneration where the patients are typically wheelchair-bound and die in their mid-twenties from cardiac or respiratory failure or both. The aim of this study was to investigate the potential of human dental pulp stem cells (hDPSCs) and human amniotic fluid stem cells (hAFSCs) to differentiate toward a skeletal myogenic lineage using several different protocols in order to determine the optimal conditions for achieving myogenic commitment and to subsequently evaluate their contribution in the improvement of the pathological features associated with dystrophic skeletal muscle when intramuscularly injected into *mdx*/SCID mice, an immune-compromised animal model of DMD.

**Methods:**

Human DPSCs and AFSCs were differentiated toward myogenic lineage in vitro through the direct co-culture with a myogenic cell line (C2C12 cells) and through a preliminary demethylation treatment with 5-Aza-2′-deoxycytidine (5-Aza), respectively. The commitment and differentiation of both hDPSCs and hAFSCs were evaluated by immunofluorescence and Western blot analysis. Subsequently, hDPSCs and hAFSCs, preliminarily demethylated and pre-differentiated toward a myogenic lineage for 2 weeks, were injected into the dystrophic gastrocnemius muscles of *mdx*/SCID mice. After 1, 2, and 4 weeks, the gastrocnemius muscles were taken for immunofluorescence and histological analyses.

**Results:**

Both populations of cells engrafted within the host muscle of *mdx*/SCID mice and through a paracrine effect promoted angiogenesis and reduced fibrosis, which eventually led to an improvement of the histopathology of the dystrophic muscle.

**Conclusion:**

This study shows that hAFSCs and hDPSCs represent potential sources of stem cells for translational strategies to improve the histopathology and potentially alleviate the muscle weakness in patients with DMD.

**Electronic supplementary material:**

The online version of this article (doi:10.1186/s13287-015-0141-y) contains supplementary material, which is available to authorized users.

## Introduction

Duchenne muscular dystrophy (DMD) is an X-linked recessive genetic disease characterized by the lack of the structural protein, dystrophin, which is the primary component of the dystrophin-glycoprotein complex (DGC) that links the cytoskeleton to the extracellular matrix, thus providing stability to the sarcolemma during muscle contraction [1]. DMD is one of the most common (1 in 3500 live male births) and severe of the muscular dystrophies, presenting with severe progressive degeneration of skeletal muscle fibers and resulting in the death of the patients, typically before their third decade of life [2].

The use of corticosteroids delays the loss of muscle function in patients with DMD; however, currently no therapy is capable of preventing the progressive muscle degenerative processes associated with DMD [3], although several different therapeutic approaches, including cell and gene therapies, have been tested over the years.

Cell therapy could be used to recover the lack of dystrophin in dystrophic muscles and thus repair the damaged muscle fibers and prevent future muscle degeneration. Recently, various stem cell populations have been used to treat muscular dystrophy. Although dystrophin represents the major target protein to be restored in patients with DMD, it has become clear over the last few years that an improvement of muscle histopathologies through promotion of angiogenesis and reduction of fibrosis can be achieved via a paracrine effect of the injected stem cells [4–6].

Stem cells obtained from different sources, including bone marrow, adipose tissue, umbilical cord, and placenta, have been investigated and could represent alternative tissue sources for obtaining adult multi-lineage progenitor cells [7–10]. Human dental pulp stem cells (hDPSCs) have been identified and can be harvested from human dental pulp during routine extraction of either deciduous teeth or third molars. hDPSCs have been widely demonstrated to be clonogenic cells capable of both self-renewal and multiple lineage differentiation [11–13]. Human amniotic fluid stem cells (hAFSCs) have also been identified and constitute approximately 1 % of the cells that could potentially be obtained from human amniocentesis backup specimens [14], samples that would otherwise be discarded. hAFSCs have been shown to possess self-renewal and multi-differentiation capacities, do not induce or form teratomas, and are far less controversial than embryonic stem cells [14, 15].

In this study, we investigated the potential of hDPSCs and hAFSCs to differentiate toward a skeletal myogenic lineage by using several different protocols in order to determine the optimal conditions for achieving myogenic commitment. The first protocol was based on the direct co-culture of the human cells with murine myoblasts in order to evaluate the capacity of both populations to fuse and form mature myotubes when directly co-cultured with mouse myoblasts.

The second protocol was based on the use of the demethylating agent 5-Aza-2′-deoxycytidine (5-Aza) and supplementation of the cultures with or without the addition of conditioned medium (CM) from differentiated mouse myoblast cultures, medium that contains numerous soluble factors that can promote myogenic differentiation, including insulin-like growth factor-II (IGF-II) [16]. To verify the effective differentiation of the hDPSCs and hAFSCs toward a skeletal myogenic lineage, we investigated the expression of muscle-specific factors and markers, including myogenin, myosin heavy chain (MyHC), and desmin.

It has been previously established that genes related to myogenic differentiation are controlled by DNA methylation and that the use of the demethylating agent 5-Aza was able to induce adult human bone marrow stem cells to differentiate toward a cardiomyogenic lineage [17, 18]. Also, demethylating agent 5-Aza was found able to increase the myogenic potential of muscle cells [19]. After treatment with 5-Aza and pre-differentiation in vitro, we tested the ability of the hDPSCs and hAFSCs to restore dystrophin expression and contribute to the amelioration of the pathological features associated with dystrophic skeletal muscle when intramuscularly injected into *mdx*/SCID mice, an immune-compromised animal model of DMD.

## Methods

### Cell culture and sorting

Human dental pulp was extracted from the enclosed third molar of teenage subjects undergoing routine tooth extractions, following written informed consent from their parents. All procedures for collecting the human samples were approved by the Provincial Ethics Committee of Santa Maria Nuova Hospital of Reggio Emilia. hDPSCs were isolated from dental pulp, as previously described by Riccio et al. [20]. The STRO-1^+^ hDPSCs subpopulation of cells was obtained via magnetic-activated cell sorting (MACS) (Miltenyi Biotec, Bergisch Gladbach, Germany), using a mouse anti-STRO-1 antibody (Ab) (Santa Cruz Biotechnology, Inc., Dallas, TX, USA).

Supernumerary amniocentesis samples were provided by Laboratorio di Genetica, Ospedale Santa Maria Nuova (Reggio Emilia, Italy). All the samples were collected after informed consent was obtained from the patients in accordance with Italian law and ethical committee guidelines. hAFSCs were isolated as previously described by De Coppi et al. [14]. Briefly, human amniocentesis cultures were harvested by trypsinization and immunoselected by MACS by using a rabbit anti-c-Kit Ab (Santa Cruz Biotechnology, Inc.). Both cell populations were expanded in culture medium containing α-MEM (alpha-minimum essential medium) plus 20 % fetal calf serum (FCS), 2 mM L-glutamine, 100 U/ml penicillin, and 100 μg/ml streptomycin (Sigma-Aldrich, St. Louis, MO, USA) and incubated at 37 °C in an atmosphere of 5 % CO_2_.

The expression of STRO-1 and c-Kit antigens was assessed through immunofluorescent staining, as described below. Moreover, the expression of STRO-1 and c-Kit antigens by hDPSCs and hAFSCs, respectively, was evaluated through fluorescence-activated cell sorting (FACS) analysis by indirect staining using mouse anti-STRO-1 IgM and rabbit anti-c-Kit IgG (Santa Cruz Biotechnology, Inc.) followed by goat anti-mouse-IgG-Alexa488 and goat anti-rabbit IgG-Alexa488 (Invitrogen, part of Thermo Fisher Scientific, Waltham, MA, USA). Non-specific fluorescence was assessed by using normal mouse IgG or IgM followed by the secondary Ab as described above. Samples were analyzed by using a 16-parameter CyFlow ML flow cytometer (Partec GmbH, Munster, Germany), equipped with a 488-nm blue solid-state, a 635-nm red diode laser, an ultraviolet mercury lamp HBO, a 532-nm green solid-state laser, a 405-nm violet laser, and a charge-coupled device camera. Data were acquired in list mode by using FloMax (Partec GmbH) software and then analyzed by FlowJo 9.4.11 (Treestar Inc., now part of FlowJo LLC, Ashland, OR, USA) under MacOS 10.

### Animals

Eight- to ten-week-old male B10ScSn.Cg-*Prkdc*^*scid*^*Dmd*^*mdx*^/J (*mdx*/SCID; The Jackson Laboratory, Bar Harbor, ME, USA) mice were housed in the animal facility located in the Bridgeside Point II building, University of Pittsburgh, under stable conditions of humidity and temperature in a controlled vivarium on a 12:12 h light-dark cycle with free access to food and water. All of the animal experiments were performed following approval by the Institutional Animal Care and Use Committee (IACUC) of the University of Pittsburgh.

### Myogenic differentiation in vitro

To evaluate the myogenic potential of the STRO-1^+^ hDPSCs and c-Kit^+^ hAFSCs, the cells underwent distinct differentiation protocols. The first protocol required the direct co-culture of the hDPSCs or hAFSCs with the C2C12 mouse myoblast cell line as previously described by Pisciotta et al. [21]. Human stem cells and mouse myoblasts were seeded on coverslips at a ratio of 10:1 and maintained in expansion medium containing DMEM high glucose (DMEM-HG) plus 10 % FCS, 2 mM L-glutamine, 100 U/ml penicillin, and 100 μg/ml streptomycin, until the cells reached confluency. At this point, the expansion medium was replaced with fusion medium, containing DMEM-HG plus 1 % FCS, 2 mM L-glutamine, 100 U/ml penicillin, 100 μg/ml streptomycin, and 10 nM insulin and continued to be co-cultured in fusion medium for 14 days.

The second protocol involved the myogenic differentiation of hDPSCs and hAFSCs by first treating the cells with 10 μM 5-Aza without direct co-culture. hDPSCs and hAFSCs were seeded on coverslips at a cell density of 4000 cells/cm^2^ in expansion medium (DMEM-HG plus 10 % FCS, 2 mM L-glutamine, 100 U/ml penicillin, and 100 μg/ml streptomycin), and upon reaching confluency the medium was replaced with DMEM low glucose (DMEM-LG), plus 10 % horse serum, 0.5 % chicken serum, 2 mM L-glutamine, 100 U/ml penicillin, and 100 μg/ml streptomycin, supplemented with 10 μM 5-Aza for 24 h. Subsequently, cells were rinsed twice in phosphate-buffered saline (PBS) and kept in myogenic medium containing DMEM-LG, plus 5 % horse serum, 0.5 % chicken serum, 2 mM L-glutamine, 100 U/ml penicillin, 100 μg/ml streptomycin, and 10 nM insulin for 24 h. The following day, the horse serum concentration in the myogenic medium was reduced to 2 %. Half of the hDPSCs and hAFSCs were differentiated under these conditions while the other half underwent the same treatment with the addition of CM from differentiated C2C12 mouse myoblast cells.

### Western Blot

To further evaluate the in vitro differentiation capacities of the STRO-1^+^ hDPSCs and c-Kit^+^ hAFSCs after their induction toward a myogenic lineage using the protocols outlined above, Western blot (WB) analysis was performed. Whole cell lysates were obtained at 2 weeks after initiating differentiation and processed as previously described by Pisciotta et al. [21]. Thirty micrograms of protein extract, quantified by a Bradford Protein Assay (Bio-Rad Laboratories, Hercules, CA, USA), underwent SDS-polyacrylamide gel electrophoresis and were transferred to PVDF membranes. The following Abs were used: mouse anti-myogenin and rabbit anti-desmin (Sigma-Aldrich), all diluted 1:1000. Peroxidase-labelled anti-mouse (diluted 1:2000) and anti-rabbit (diluted 1:3000) secondary Abs were used. Whole cell lysate obtained from C2C12 myoblasts was used as a positive control. The membranes were visualized by using enhanced chemioluminescence (Amersham, now part of GE Healthcare, Little Chalfont, UK). Anti-actin Ab was used as control of protein loading. Densitometry of the bands was performed by using ImageJ analysis software (National Institutes of Health, Bethesda, MD, USA). Data were then normalized to values of background and the control actin band.

### In vivo transplantation of the hDPSCs and hAFSCs into *mdx*/SCID mice

To evaluate whether the hDPSCs and hAFSCs were capable of contributing to the regeneration of dystrophin-expressing myofibers, both human stem cell populations demethylated with 10 μM 5-Aza and pre-differentiated in vitro for 2 weeks in myogenic medium, with and without the addition of CM from the differentiated C2C12 cells, were transplanted into the gastrocnemius muscles (GMs) of *mdx*/SCID mice. Briefly, pre-differentiated hDPSCs and hAFSCs (5 × 10^5^), re-suspended in 30 μl of PBS, were injected into the GMs of male *mdx*/SCID mice that were 8–10 weeks of age. Each animal received two injections: in the left GM, the cells pre-differentiated after demethylating treatment; in the right GM, the cells pre-differentiated after demethylating treatment and the addition of CM. Non-injected muscles were used as negative controls. The animals were euthanized at 7, 14, and 28 days after cell transplantation, and the GMs were harvested and then snap-frozen in liquid nitrogen-cooled 2-methylbutane. Subsequently, transverse serial sections (8 μm thick) of the frozen muscles were cut by using an H525 MICROM cryostat (Thermo Fisher Scientific). Sections were collected on Super Frost Plus slides (Thermo Fisher Scientific) and then stored at −80 °C. In total, 30 animals were used in this study (controls n = 6; treated n = 24). Animal procedures were performed in compliance with the guidelines approved by the IACUC of the University of Pittsburgh (Pittsburgh, PA, USA).

### Immunofluorescent microscopy and histology

hDPSCs and hAFSCs were seeded on coverslips following MACS for STRO-1 and c-Kit, respectively. Once adhered, the cells were fixed with 4 % paraformaldehyde, blocked with 3 % bovine serum albumin (BSA) in PBS for 30 min at room temperature, and incubated with mouse anti-STRO-1 and rabbit anti-c-Kit primary Abs (Santa Cruz Biotechnology, Inc.) diluted 1:50 in PBS containing 3 % BSA for 1 h at room temperature. After washing in PBS containing 3 % BSA, the samples were incubated for 1 h at room temperature with the secondary Abs diluted 1:200 in PBS containing 3 % BSA (goat anti-mouse Alexa546 and goat anti-rabbit Alexa488; Invitrogen). After washing in PBS, samples were stained with 1 μg/ml 4′,6-diamidino-2-phenylindole (DAPI) in PBS for 1 min and then mounted with anti-fade medium (VectaMount AQ Aqueous Mounting Medium; Vector Laboratories, Burlingame, CA, USA). Negative controls consisted of samples not incubated with the primary Ab. Immunofluorescence analysis was performed by using a Nikon A1 confocal laser scanning microscope (Nikon, Tokyo, Japan) as described by Riccio et al. [20]. The confocal serial sections were processed with ImageJ software to obtain three-dimensional projections, and image rendering was performed by using Adobe Photoshop Software (Adobe Systems Incorporated, San Jose, CA, USA).

Fixed monolayer cells, differentiated in vitro on coverslips, were permeabilized with 0.1 % Triton X-100 in PBS for 5 min and then blocked with 3 % BSA in PBS for 30 min at room temperature. The following primary Abs were used: mouse anti-human nuclei (hNu) (EMD Millipore, Billerica, MA, USA), mouse anti-myogenin, rabbit anti-MyHC, and rabbit anti-desmin (all from Sigma-Aldrich). All the primary Abs were diluted 1:50 in PBS containing 3 % BSA and incubated for 1 h at room temperature. Secondary Abs were diluted 1:200 in PBS containing 3 % BSA and incubated for 1 h at room temperature (goat anti-mouse Alexa488 and donkey anti-rabbit Alexa594; Invitrogen); after washing in PBS, the cells were counterstained with 1 μg/ml DAPI in PBS for 3 min and then mounted with anti-fade medium (VectaMount AQ Aqueous Mounting Medium; Vector Laboratories). Negative controls consisted of samples not incubated with the primary Ab.

Histological unfixed sections from frozen muscle were processed as described above. Mouse anti-human mitochondrial protein (hMit) (1:80; EMD Millipore), rabbit anti-human dystrophin (1:500, kindly provided by Bing Wang, University of Pittsburgh), and rabbit anti-human von Willebrand factor (hvWill) (1:100; EMD Millipore) were used as primary Abs. The following secondary Abs were used at a 1:200 dilution: donkey anti-mouse Alexa488, donkey anti-rabbit Alexa594, donkey anti-mouse Alexa594, and goat anti-rabbit Alexa488 (Invitrogen). Samples not incubated with primary Abs were used as negative controls. Immunofluorescence analysis was performed on a Nikon Eclipse E800 microscope (Nikon) by using Northern Eclipse imaging software (EMPIX Imaging) to capture images. The image rendering was performed by using Adobe Photoshop software.

Routine hematoxylin and eosin (H&E) staining was performed on serial cross-sections (8 μm) of frozen GMs in order to analyze the morphological details of the tissues. Images of the entire muscle section were captured, the number of centrally nucleated myofibers was determined, and the percentage of centrally nucleated fibers to all myofibers in the entire muscle section was calculated. Likewise, the average sizes of centrally nucleated fibers and whole muscle fibers were measured.

The extent of fibrosis in the muscle cryosections was determined by using Masson’s trichrome staining to detect the collagen content of the muscles, as detailed in the protocol of the manufacturer (Masson’s Trichrome stain kit; IMEB, Inc., San Marcos, CA, USA). Images of the histological samples were obtained with a Nikon Eclipse E800 microscope.

The extent of angiogenesis in the injected muscle cryosections was determined by quantifying the number of blood vessels (*vasa*) positively stained by anti-hvWill Ab. Values, calculated on three different cryosections of each of the six samples per experimental group, were expressed as mean ± standard deviation.

### Statistical analysis

Data were expressed as the mean ± standard deviation. Differences between experimental groups consisting of 5–6 samples each were analyzed by analysis-of-variance test followed by Tukey’s Multiple Comparison Test (GraphPad Prism Software version 5; GraphPad Software, Inc., San Diego, CA, USA). In all analyses, *P* values of less than 0.05 were considered statistically significant.

## Results

### Cell sorting

**Fig. 1:**
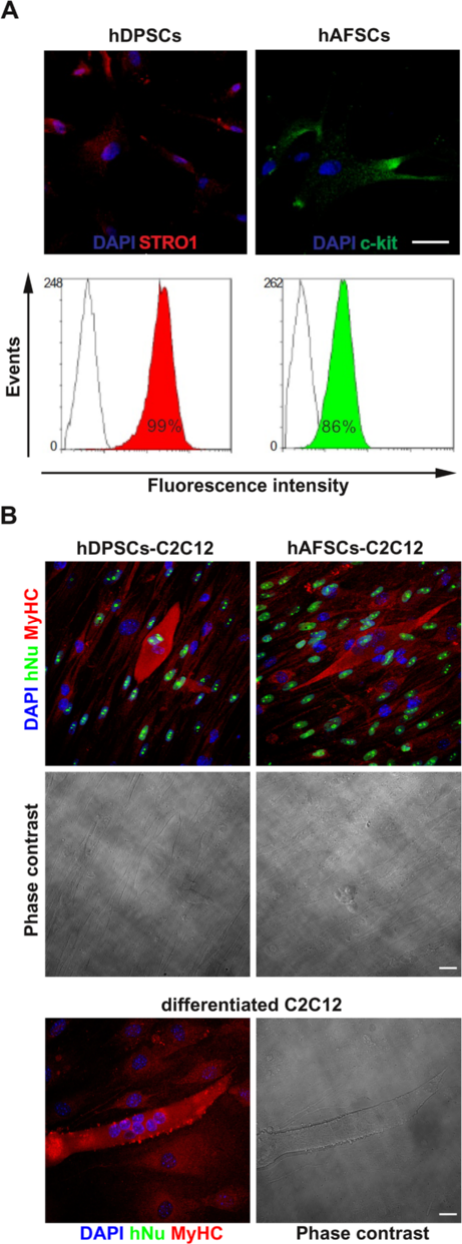
Cell characterization after cell sorting and evaluation of direct co-culture with C2C12 mouse myoblasts. **a** Cell characterization after MACS. On the *top*, fluorescent images of hDPSCs and hAFSCs positively labeled with anti-STRO-1 (red) and anti-c-Kit (green) antibodies, respectively. At the *bottom*, flow cytofluorimetric analysis of hDPSCs and hAFSCs sorted by MACS; the percentage of positive cells is indicated. **b** Evaluation of in vitro direct co-culture of hDPSCs and hAFSCs with C2C12 mouse myoblasts. Triple immunofluorescent staining of hAFSCs and hDPSCs co-cultured with C2C12 mouse myoblasts revealed newly formed myotubes which are labeled with DAPI (blue), anti-hNu (green), and anti-MyHC (red) antibodies. C2C12 cells differentiated alone were used as control. Scale bars = 50 μm. *DAPI* 4′,6-diamidino-2-phenylindole, *hAFSC* human amniotic fluid stem cell, *hDPSC* human dental pulp stem cell, *hNu* human nuclei, *MACS* magnetic-activated cell sorting, *MyHC* myosin heavy chain

To verify whether hDPSCs and hAFSCs previously sorted by MACS were STRO-1^+^ and c-Kit^+^, respectively, immunocytochemistry and flow cytometry were performed (Fig. 1a). Immunocytochemical analysis showed that the sorted cells were positive for their respective surface antigens (Fig. 1a, top). Moreover, flow cytometry demonstrated that almost all of the sorted cells were positive for STRO-1 and c-Kit, respectively (Fig. 1a, bottom).

### Myogenic differentiation in vitro

To test the cell populations’ myogenic potential, the hDPSCs and hAFSCs were induced to differentiate into a myogenic lineage in vitro. The immunofluorescent analysis performed on the hDPSCs and hAFSCs after 14 days of direct co-culture with C2C12 cells revealed the formation of myotubes that were positive for both anti-hNu and anti-MyHC Abs, as shown in Fig. 1b (top). In particular, newly formed hybrid myotubes that contained both human and mouse nuclei were observed. As expected, C2C12 cells differentiated alone did not show any positive staining to anti-hNu Ab (Fig. 1b, bottom).

**Fig. 2:**
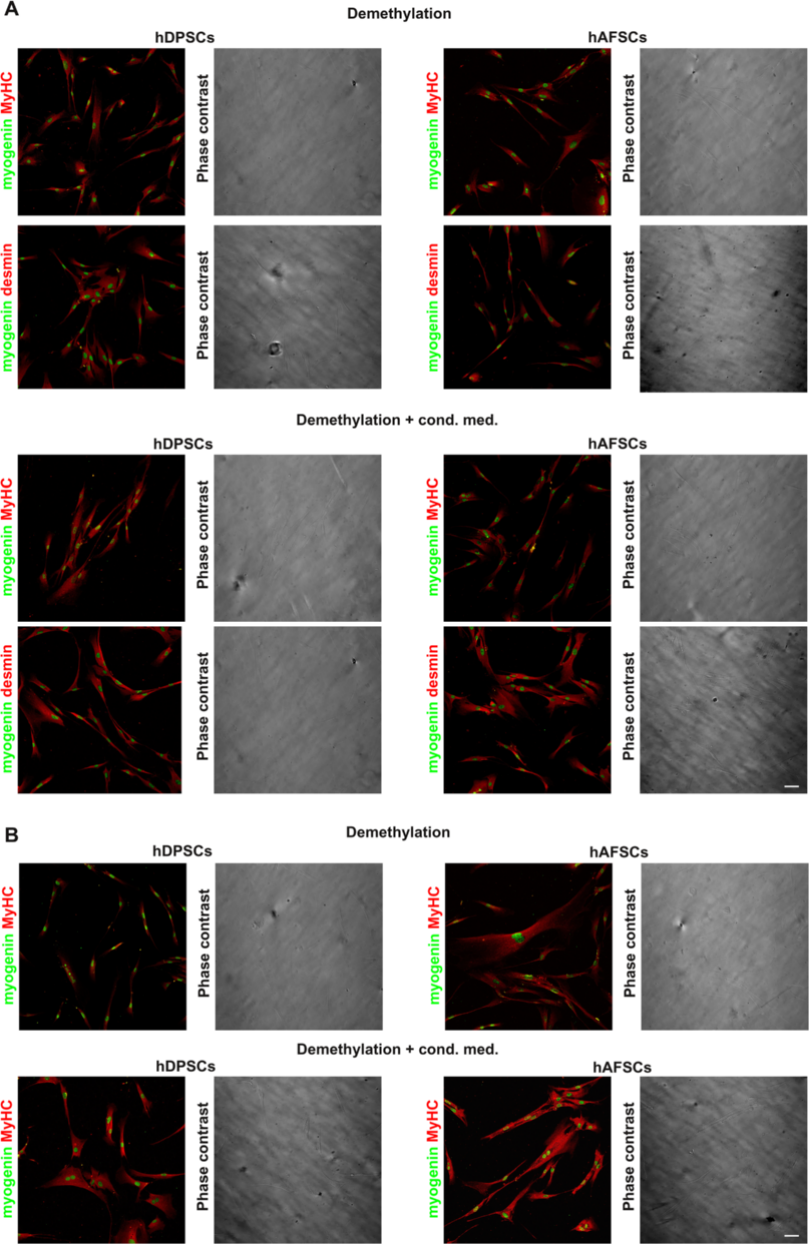
Immunofluorescent analysis after 2 and 4 weeks of myogenic differentiation of hDPSCs and hAFSCs by demethylation treatment. Immunofluorescent staining with anti-myogenin (green)/anti-MyHC (red) and anti-myogenin (green)/anti-desmin (red) antibodies. Myogenic differentiation was induced with and without the addition of CM from differentiated C2C12 cultures. Human DPSCs and AFSCs underwent myogenic commitment as early as after 14 days of induction (**a**) by expressing the muscle-specific markers myogenin, MyHC, and desmin. However, multinucleated myotube formation occurred only after 4 weeks of induction (**b**). Scale bars = 50 μm. *CM* conditioned medium, *hAFSC* human amniotic fluid stem cell, *hDPSC* human dental pulp stem cell, *MyHC* myosin heavy chain

Immunofluorescent labeling was also carried out on the hDPSCs and hAFSCs differentiated alone, following DNA demethylation treatment with 5-Aza (Fig. 2). After 14 days of induction, the hDPSCs expressed myogenin, which is an early marker for the entry of myoblasts into the differentiation pathway; moreover, they expressed MyHC and desmin, which are late-myogenic markers. The hAFSCs also underwent myogenic commitment as demonstrated by their positive staining for myogenin, MyHC, and desmin, which confirmed the terminal myogenic commitment of the cells (Fig. 2a). Similar results were observed for both the hDPSCs and hAFSCs differentiated after demethylation with the addition of CM from the differentiated C2C12 cell cultures (Fig. 2a). After 2 weeks of induction, no myotube formation was detected; however, after 4 weeks, myotube formation could be detected in both the hDPSC and hAFSC cultures (Fig. 2b). hDPSCs and hAFSCs cultured in myogenic induction medium, but not undergoing preliminary demethylation treatment, did not show any labeling for the myogenic-specific markers myogenin, MyHC, or desmin (Additional file 1: Fig. S1).

**Fig. 3:**
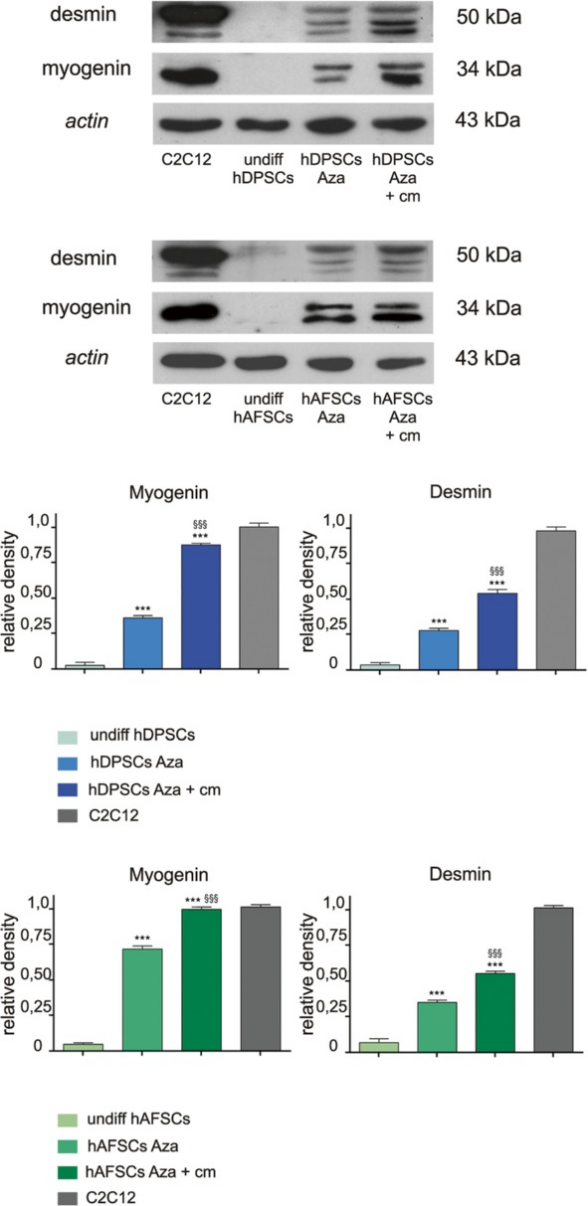
Western blot analysis of muscle-specific markers in differentiated hDPSCs and hAFSCs. Western blot analysis of myogenin and desmin expression in whole cell lysates of differentiated hDPSCs and hAFSCs with (hDPSCs Aza + cm, hAFSCs Aza + cm) and without the addition of C2C12 CM (hDPSCs Aza, hAFSCs Aza). Whole cell lysates were collected from three plates for each experimental group. Actin bands demonstrate that an equal amount of protein was loaded in each lane. Densitometric analysis of Western blot bands is shown at the bottom (analysis of variance followed by Tukey’s test: ***P < 0.001 hDPSCs Aza and hDPSCs Aza + cm versus undiff hDPSCs; ***P < 0.001 hAFSCs Aza and hAFSCs Aza + cm versus undiff hAFSCs; ^§§§^ *P* < 0.001 hDPSCs Aza + cm versus hDPSCs Aza; ^§§§^ *P* < 0.001 hAFSCs Aza + cm versus hAFSCs Aza). *CM* conditioned medium, *hAFSC* human amniotic fluid stem cell, *hDPSC* human dental pulp stem cell

To verify the commitment and differentiation of the hDPSCs and hAFSCs toward a myogenic lineage, the expression of myogenin and desmin was also evaluated by WB analysis by using whole cell lysates from the hDPSCs and hAFSCs treated with 5-Aza and treated or not treated with C2C12 CM (Fig. 3, top). A densitometric analysis was carried out on the WB bands to obtain a semi-quantitative analysis of the protein amount (Fig. 3, bottom). Both the hDPSCs and hAFSCs expressed the muscle-specific markers myogenin and desmin after 2 weeks of myogenic induction as revealed by the existence of the specific protein bands corresponding to 34 and 50 kDa, respectively (Fig. 3). Densitometric analysis revealed that, in both the differentiated hDPSCs and hAFSCs, both with and without being treated with the C2C12 CM, there was a significant expression of the myogenic markers compared with the controls (***P <0.001). In particular, a significantly higher expression of both myogenin- and desmin-positive cells were observed in the hDPSC and hAFSC cultures differentiated via 5-Aza with the addition of C2C12 CM than in the cultures not treated with C2C12 CM, respectively (^§§§^*P* <0.001).

### Engraftment evaluation and muscle regeneration after in vivo transplantation

**Fig. 4:**
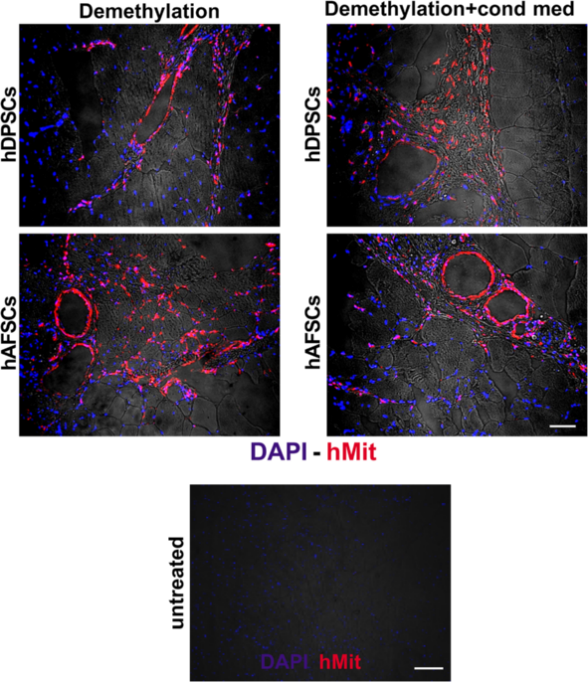
Engraftment evaluation 7 days after cell injection in *mdx*/SCID GMs. Immunofluorescent staining with DAPI (blue) and anti-hMit antibody (red) shows the presence of human DPSCs and AFSCs within the host dystrophic muscle, after the cell injection, following both differentiation conditions. Untreated samples, consisting of non-injected *mdx*/SCID GMs, were used as negative controls. Scale bars = 50 μm. *hAFSC*, human amniotic fluid stem cell, *DAPI* 4′,6-diamidino-2-phenylindole, *hDPSC*, human dental pulp stem cell, *GM* gastrocnemius muscle, *hMit* human mitochondrial protein

**Fig. 5:**
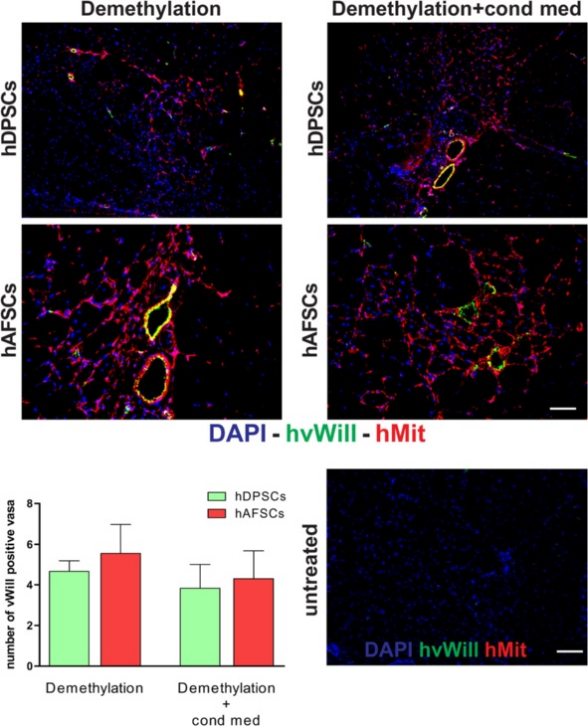
Evaluation of angiogenesis promotion 7 days after cell injection into *mdx*/SCID GMs. Immunofluorescent labeling with DAPI (blue), anti-hvWill (green), and anti-hMit (red) antibodies. Green and red signals appear co-localized, showing that hDPSCs and hAFSCs are present within the endothelium of new *vasa*. The number of vessels positive for hvWill, calculated on three different cryosections of the six samples per experimental group, is expressed as mean ± standard deviation. Untreated samples, consisting of non-injected *mdx*/SCID GMs, were used as negative controls. Scale bars = 50 μm. *DAPI* 4′,6-diamidino-2-phenylindole, *GM* gastrocnemius muscle, *hAFSC* human amniotic fluid stem cell, *hDPSC* human dental pulp stem cell, *hMit* human mitochondrial protein, *hvWill* human von Willebrand factor

**Fig. 6:**
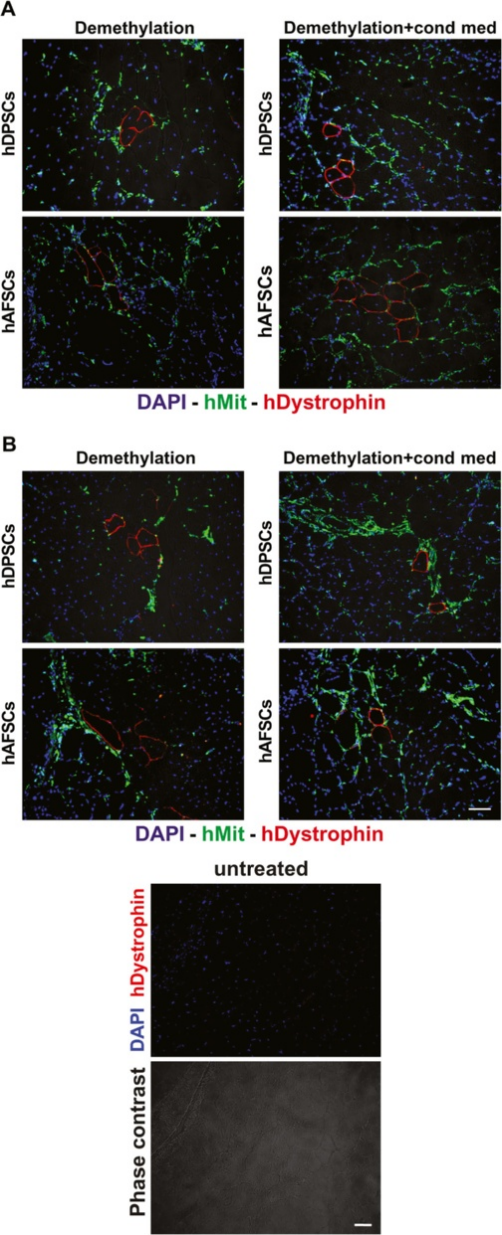
Evaluation of dystrophin expression after cell injection in *mdx*/SCID GMs. **a** Immunofluorescent staining with DAPI (blue), anti-hMit (green), and anti-hDystrophin (red) antibodies shows the presence of human DPSCs and AFSCs within human dystrophin-positive muscle fibers 14 days after cell injection. **b** Dystrophin-expressing muscle fibers were also observed at 28 days post-injection. Untreated samples, consisting of non-injected *mdx*/SCID GMs, were used as negative controls. Scale bars = 50 μm. *hAFSC*, human amniotic fluid stem cell, *DAPI* 4′,6-diamidino-2-phenylindole, *hDPSC*, human dental pulp stem cell, *GM* gastrocnemius muscle, *hDystrophin* human Dystrophin

hDPSCs and hAFSCs that were pre-differentiated for 2 weeks toward a myogenic lineage, via 5-Aza demethylation, with and without treatment with C2C12 CM, were injected into the GMs of *mdx*/SCID mice to test their ability to regenerate dystrophin-expressing muscle fibers within the dystrophic skeletal muscle of *mdx*/SCID mice. The injected animals were sacrificed at different time points, and the GMs were flash-frozen and processed for further analyses. Immunofluorescent analysis showed that as early as 7 days after injection the hDPSCs and hAFSCs could be detected histologically within the host muscle, as demonstrated by the positive staining of human mitochondrial protein. Similar results were observed with or without culturing the cells in C2C12 CM (Fig. 4). Double immunofluorescent staining, performed 7 days after cell injection by using anti-hMit and anti-hvWill Abs, revealed the presence of human cells positively stained as endothelial cells within the blood vessel walls (Fig. 5). Quantification of *vasa* positively stained by anti-hvWill Ab did not show any significant differences between the experimental groups (Fig. 5, histograms). Untreated samples, consisting of non-injected GMs, did not show any positive staining against hMit and hvWill Abs (Figs. 4 and 5). Moreover, 2 weeks after cell injection, the immunofluorescent labelling showed the presence of dystrophin-expressing myofibers within the injected dystrophic skeletal muscle. In particular, the myofibers expressing dystrophin were also positively stained for human mitochondrial protein (Fig. 6). Similar results were observed for both pre-differentiation conditions (Fig. 6a). Restored expression of dystrophin by the mouse muscle fibers positively labelled for anti-human mitochondria Ab was still detectable 4 weeks after cell injection (Fig. 6b). Again, similar results were observed by using both pre-differentiation conditions (Fig. 6a, b). The number of dystrophin-positive myofibers was very limited after the injection of either hDPSCs and hAFSCs; therefore, they could resemble revertant myofibers.

### Histological and histomorphometric analyses

**Fig. 7:**
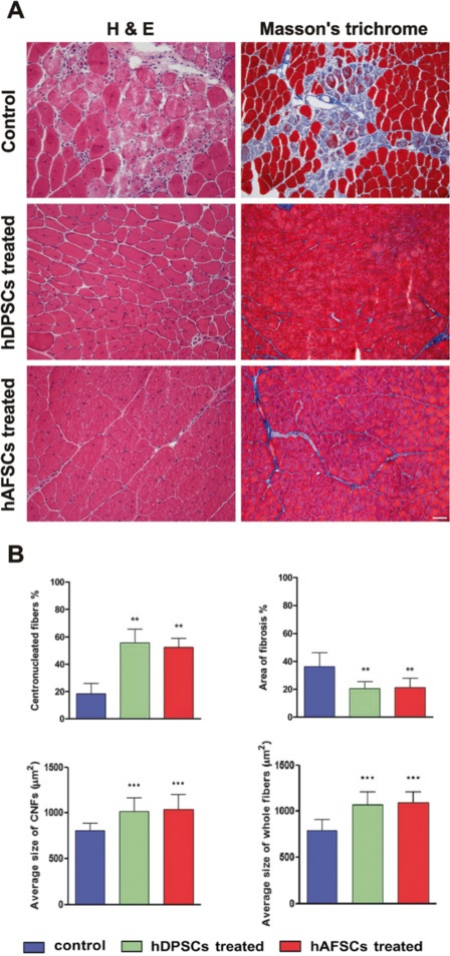
Histological analyses of the injected and non-injected *mdx*/SCID skeletal muscles. **a** H&E staining shows active muscle regeneration within the muscles treated with human DPSCs and AFSCs, as represented by a high number of centronucleated muscle fibers. Comparison of fibrotic processes between controls (non-injected *mdx*/SCID GMs) and *mdx*/SCID GMs injected with human DPSCs and AFSCs. Masson’s trichrome staining indicates a reduction in fibrosis within the muscles treated with human DPSCs and AFSCs, when compared with controls (non-injected muscle). Scale bar = 50 μm. **b** Histomorphometric analysis of the muscles treated with human DPSCs and AFSCs. *Upper left*: histograms represent the percentage of regenerating centronucleated muscle fibers. **P <0.01 versus control (ANOVA followed by Tukey’s test). *Upper right*: histograms represent the percentage area of fibrosis. **P <0.01 versus control (ANOVA followed by Tukey’s test). *Lower left*: histograms represent the average size (in square micrometers) of centrally nucleated muscle fibers. ***P <0.001 versus control (ANOVA followed by Tukey’s test). *Lower right*: histograms represent average size (in square micrometers) of whole muscle fibers. ***P <0.001 versus control (ANOVA followed by Tukey’s test). *hAFSC*, human amniotic fluid stem cell, *ANOVA* analysis of variance, *hDPSC*, human dental pulp stem cell, *GM* gastrocnemius muscle, *H&E* hematoxylin and eosin

To evaluate the presence of fibrous tissue within the dystrophic skeletal muscle of the *mdx*/SCID mice after cell injection, serial sections from GMs harvested at 4 weeks after injection were stained with H&E and Masson’s trichrome. Histological analysis of H&E staining revealed that the muscles injected with human stem cells contained areas of muscle regeneration, as demonstrated by the presence of centronucleated muscle fibers (Fig. 7a). Particularly, the percentages of centronucleated muscle fibers in the GMs treated with hDPSCs and hAFSCs were significantly higher when compared with the non-injected (control) GM group (Fig. 7b, upper left).

Similarly, analysis of Masson’s trichrome staining in Fig. 7a shows a reduction in fibrosis within the skeletal muscle injected with the pre-differentiated hDPSCs and hAFSCs, compared with the controls. In particular, the GMs treated with hDPSCs and hAFSCs showed a significant reduction (*P* < 0.01) in fibrotic areas in comparison with the non-injected (control) GMs (Fig. 7b, upper right).

Moreover, histomorphometric analysis revealed that the average sizes (in square micrometers) of centrally nucleated fibers were significantly higher in GMs treated with hDPSCs and hAFSCs compared with the non-injected control GMs (Fig. 7b, lower left). Similarly, even the average sizes of whole muscle fibers in GMs treated with hDPSCs and hAFSCs were significantly higher compared with the non-injected controls (Fig. 7b, lower right).

## Discussion

The muscular dystrophies are a diverse set of genetic myopathies characterized by progressive muscle weakness, atrophy, and the replacement of healthy myofibers with fat and scar tissue [22, 23]. Several forms of these diseases are caused by mutations in components of the dystrophin-glycoprotein complex (DGC), which links the actin cytoskeleton of the myofibers to the extracellular matrix and is a fundamental component required to maintain the contractile structure of the skeletal muscle [24, 25]. Mutations that cause disruptions in the component proteins of the DGC lead to a series of different myopathies, of which DMD is both the most severe and the most common, affecting 1 in 3500 live male births. Homologues of DMD have been identified in several animals, such as the *dy/dy* mouse, the extensively studied *mdx* mouse, the *mdx/utrn*^*−/−*^ (dystrophin- and utrophin-deficient) mouse, and the muscular dystrophic golden retriever dog [26]. Although there is no definitive model of DMD at this time, the variety of DMD homologues available is adequate for understanding the fundamental pathophysiology of the disease [26].

Satellite cells (SCs) can keep up with muscle fiber loss in the early stages of DMD because they can regenerate the degenerated muscle fibers, and this leads to a mild dystrophic phenotype. However, as previously demonstrated by different research groups, the pool of SCs becomes exhausted as a result of high demands for muscle regeneration and poor compensatory mechanisms, and this results in fibrous and fatty connective tissue infiltration and then in muscle weakness [27–29].

Cell therapy has been considered as a potential therapeutic treatment for DMD over the years. Partridge et al. [30] originally demonstrated that donor myoblasts could fuse with each other or host myoblasts, suggesting the possibility of functional restoration of dystrophin in defective muscle fibers.

The pivotal findings that donor heterologous myoblasts could restore dystrophin expression in the dystrophin-deficient *mdx* mouse [31] laid the foundation for a number of human clinical trials in DMD patients in the 1990s [32]. Initial clinical trials with allogeneic myoblast injection into the muscles of non-immunosuppressed DMD patients showed only a transient restoration of dystrophin-positive fibers and limited improvements in muscle strength because of a rapid cell death and immune rejection of the injected cells [33–38]. The limitations of these therapeutic approaches have triggered extensive research over the years. One of the approaches attempted the transplantation of bone marrow mesenchymal stem cells (MSCs) into the dystrophic muscles of *mdx* mice which showed that the MSCs indeed possessed the capacity to differentiate, at least in part, into muscle tissue [7, 39, 40]. Besides bone marrow MSCs, several cell types have been identified as potential sources for muscle tissue regeneration [41–44].

In the present study, we evaluated the myogenic potential of STRO-1^+^-enriched hDPSCs and c-Kit^+^-enriched hAFSCs. It has been well demonstrated that, in the dental pulp, STRO-1^+^ stem cells are capable of differentiating toward multilineage cell types with respect to non-sorted cells and this is likely due to their more homogeneous nature [45]. Similarly, as described by De Coppi et al. [14] and Bai et al. [46], c-Kit^+^ cells represent a group of purified MSCs newly found in amniotic fluid that have broad multipotency capacity.

Via two different protocols, this study aimed to determine the optimal conditions for achieving the myogenic commitment of these two populations of human stem cells. The results from the direct co-culture of hDPSCs and hAFSCs with C2C12 mouse myoblasts demonstrated that these human stem cells were capable of fusing with mouse myoblasts to form hybrid myotubes, as shown by the hNu in the myotubes through the use of anti-hNu Ab.

After being differentiated without direct co-culture by means of DNA demethylation treatment, immunofluorescence analysis revealed that hDPSCs and hAFSCs underwent myogenic commitment, even though multinucleated myotubes were not observed. Similarly, when CM from differentiated C2C12 cells was added to the myogenic medium of the demethylated cells, the expression of muscle-specific markers by the hDPSCs and hAFSCs was observed. In particular, both hDPSCs and hAFSCs reached myogenic commitment after 14 days of induction as confirmed by the cells’ expression of muscle-specific markers. Although myotube formation was not observed at the 2-week time point, by 4 weeks after the initiation of myogenic induction, myotubes were detectable, with and without the addition of C2C12 CM. This further demonstrates the ability of the hDPSCs and hAFSCs to undergo myogenic differentiation. The induction of both hDPSCs and hAFSCs toward a myogenic lineage was further demonstrated by WB analysis which showed that when CM from C2C12 cells was added to the hDPSC and hAFSC cultures, the cells’ expression of myogenic-specific markers became more pronounced. On the other hand, neither early nor late myogenic differentiation markers could be detected in the hDPSCs or hAFSCs when they were cultured in differentiation medium without the demethylating process.

These observations suggest that modulating the myogenic potential of these populations of cells could be achieved by combining demethylation (which triggers the expression of muscle regulatory factors necessary for the myogenic process to proceed) with the addition of the C2C12 conditioned fusion media, which contains soluble factors that promote myogenesis.

According to the results obtained in vitro, conditioning the cells with 5-Aza was found to be important for triggering the myogenic commitment of the hDPSCs and hAFSCs when differentiating the cells in the absence of C2C12 cells; therefore, The demethylation treatment of hDPSCs and hAFSCs with 5-Aza was performed as part of the differentiation strategy to evaluate their myogenic potential in vivo when injected into the dystrophic GMs of *mdx*/SCID mice.

Early engraftment was observed within the skeletal muscle fibers of the host, as confirmed by the presence of human cells in the injected muscles detected by staining for human mitochondria, which demonstrated that some of these injected cells were able to survive and integrate into the host muscle after transplantation. In particular, hDPSCs and hAFSCs showed that they were capable of supporting the improvement of skeletal muscle by promoting neo-angiogenesis within the transplanted muscles, which is a key player in achieving good physiological repair of the tissue [47]. In fact, according to Biscetti et al., dystrophin expression is not limited to skeletal muscle cells but is also expressed by several other cell types, including endothelial cells [48], which are known to interact directly and indirectly with myoblasts and promote the proliferation of myogenic precursor cells [49]. The physiological importance of dystrophin in the vascular endothelium is demonstrated by the fact that *mdx* mice display vascular abnormalities and their muscles experience ischemic conditions [50]. Recent findings showed that increasing the vasculature in patients with DMD might be capable of ameliorating the histological and functional phenotypes related to the disease and that developing an efficacious therapy for DMD would address improving both skeletal muscle regeneration and its vasculature [51]. Double-positive staining for human mitochondria and human von Willebrand factor demonstrated that both the hDPSCs and hAFSCs could be localized in the endothelium of newly generated *vasa* after their transplantation, and this supports the fact that they participated in the neo-angiogenesis of the transplanted muscle, a well-defined property of the cells that has been previously shown [6, 52, 53]. The ability of the hDPSCs and hAFSCs to regenerate dystrophin-expressing myofibers within the injected host muscles observed as early as 14 days post-injection was minimal and within the threshold of revertant myofibers (less than 1 %). Although these muscle fibers were stained by an Ab that specifically detects human dystrophin, which consequently indicated the participation of donor cells in the regeneration process, the number of human dystrophin-positive fibers was very limited and similar to revertant muscle fibers.

Moreover, another important finding of the present study was that the hDPSCs and hAFSCs contributed to a reduction in the formation of fibrosis within the dystrophic muscle, which further enhanced the regeneration process as was demonstrated by the increased number of centronucleated muscle fibers. In particular, the higher average sizes of centronucleated muscle fibers in GMs treated with hDPSCs and hAFSCs in comparison with control non-injected GMs suggest that the maturation process of the regenerated muscle fibers is more advanced in the cell-injected skeletal muscles.

These observations suggest that, once engrafted within the host muscle, hDPSCs and hAFSCs actively contributed to the amelioration of the dystrophic phenotype within the muscle by promoting angiogenesis and by reducing the deposition of fibrosis. It is important to note that muscle function was not tested in the present study, because *mdx* mice, despite being used as a genetic model of DMD, exhibit a very mild phenotype (especially at young age) in terms of muscle weakness as compared with patients with DMD [30]. The limited presence of dystrophin-positive fibers observed after cell transplantation could be attributable in part to limitations associated with xenotransplantation. Although the recipient is a SCID mouse, the inflammatory process can still limit the human stem cells to engraft and to functionally integrate in the host tissue. In addition, it has been observed that human stem cells exert, in general, a reduced ability to fuse with murine myofibers [54, 55].

## Conclusions

In light of these results, hDPSCs and hAFSCs hold promise as two potential stem cell populations that could be useful for the improvement of pathological features of dystrophic skeletal muscle tissues, including the reduction of fibrosis and the enhancement of angiogenesis in patients with DMD. These findings are aimed at paving the way for further investigations in order to optimally characterize the contributions of hDPSCs and hAFSCs to improve muscle function in DMD animal models.

## Additional file

Additional file 1: Figure S1. Human DPSCs and AFSCs not undergoing demethylation treatment did not show any expression of muscle-specific markers (myogenin, MyHC, and desmin) after culture in myogenic induction medium. Scale bar = 50 μm. *hAFSC*, human amniotic fluid stem cell, *hDPSC*, human dental pulp stem cell, *MyHC* myosin heavy chain. (TIFF 457 kb)

## Abbreviations

5-Aza: 5-Aza-2′-deoxycytidine
Ab: Antibody
BSA: Bovine serum albumin
CM: Conditioned medium
DAPI: 4′,6-diamidino-2-phenylindole
DGC: Dystrophin-glycoprotein complex
DMD: Duchenne muscular dystrophy
DMEM-HG: Dulbecco’s modified Eagle’s medium-high glucose
DMEM-LG: Dulbecco’s modified Eagle’s medium-low glucose
FCS: Foetal calf serum
GM: Gastrocnemius muscle
H&E: Hematoxylin and eosin
hAFSC: Human amniotic fluid stem cell
hDPSC: Human dental pulp stem cell
hMit: Human mitochondrial protein
hNu: Human Nuclei
hvWill: Human von Willebrand factor
IACUC: Institutional Animal Care and Use Committee
MACS: Magnetic-activated cell sorting
MSC: Mesenchymal stem cell
MyHC: Myosin heavy chain
PBS: Phosphate-buffered saline
SC: Satellite cell
WB: Western blot
