# Supplementary figures and images for: Stem cells isolated from human dental pulp and amniotic fluid improve skeletal muscle histopathology in mdx/SCID mice
Source: Stem Cell Res Ther. 2015 Aug 28;6(1):156. doi: 10.1186/s13287-015-0141-y (PMC4552417; doi:10.1186/s13287-015-0141-y)

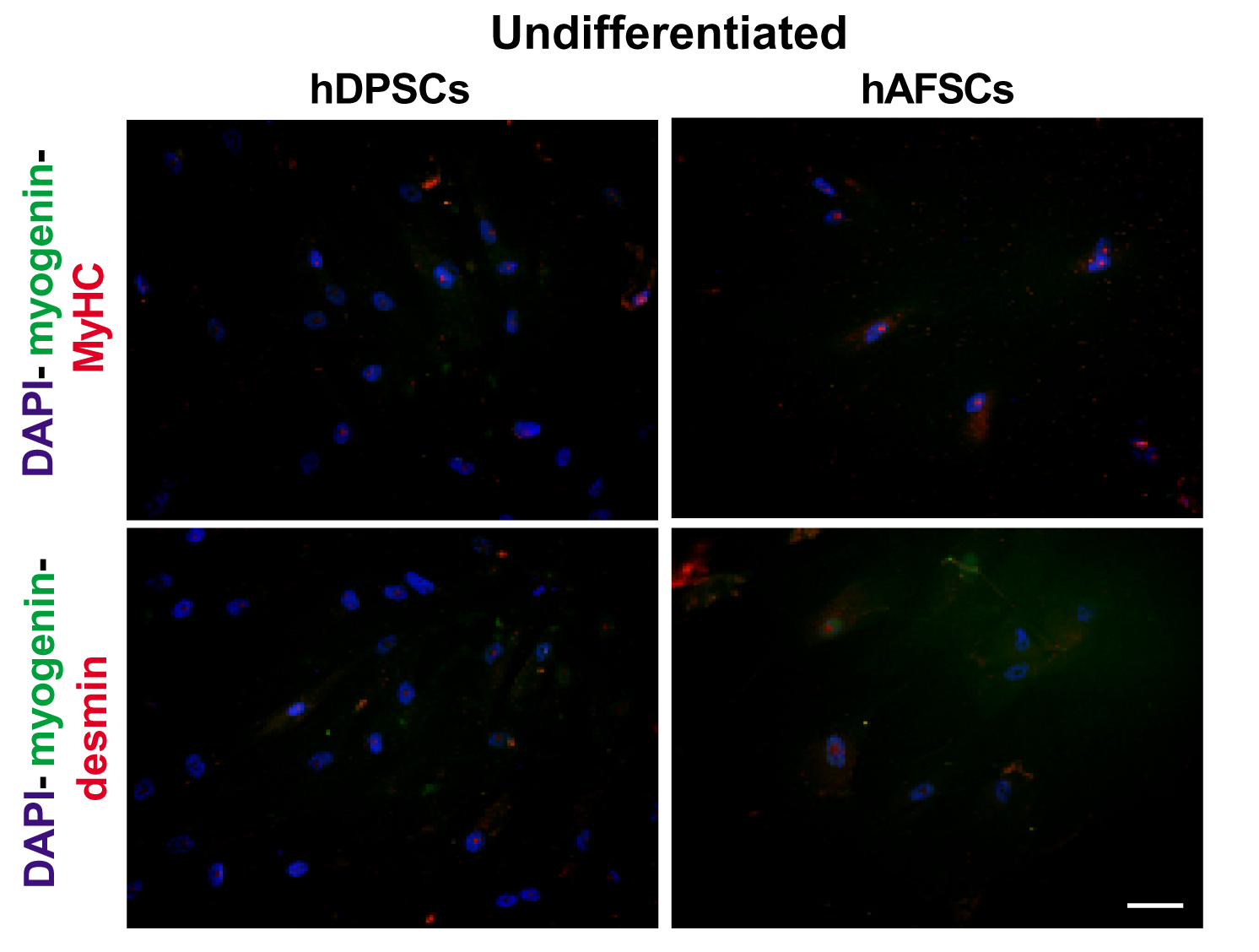

Supplement: Additional file 1: Figure S1. — Human DPSCs and AFSCs not undergoing demethylation treatment did not show any expression of muscle-specific markers (myogenin, MyHC, and desmin) after culture in myogenic induction medium. Scale bar = 50 μm. hAFSC, human amniotic fluid stem cell, hDPSC, human dental pulp stem cell, MyHC myosin heavy chain. (TIFF 457 kb) [file 13287_2015_141_MOESM1_ESM.tiff]
